# Effect of General Medical Degree Curricular Change on Mental Health of Medical Students: A Concurrent Controlled Educational Trial

**Published:** 2019-01

**Authors:** Mohammad Reza Sohrabi, Narges Malih, Hamid Reza Karimi, Zahra Hajihashemi

**Affiliations:** 1 Social Determinants of Health Research Center and Department of Community Medicine, School of Medicine, Shahid Beheshti University of Medical Sciences, Tehran, Iran.; 2 Social Determinants of Health Research Center, Shahid Beheshti University of Medical Sciences, Tehran, Iran.; 3 Department of Community Medicine, School of Medicine, Shahid Beheshti University of Medical Sciences, Tehran, Iran.; 4 Department of Rheumatology, Imam Hossein Hospital, School of Medicine, Shahid Beheshti University of Medical Sciences, Tehran, Iran.

**Keywords:** *Curriculum*, *Education*, *Health Status*, *Logistic Models*, *Medical*, *Mental Disorders*

## Abstract

**Objective:** General medical degree (GMD) curriculum usually causes significant psychological distress for medical students, especially in transition periods between preclinical, clerkship, and internship periods. This study was conducted to assess the effect of curricular change in GMD program on mental health of medical students in internship period.

**Method** **:** This study evaluated mental health of 2 concurrent groups of medical students under reformed and non-reformed GMD curriculum. In this study, 120 out of 180 interns in the non-reform GMD program and 60 interns in the reformed GMD program were selected and their mental health status evaluated using Symptom Checklist-90-Revised (SCL-90-R) questionnaire. The cut-off point of 0.7 was used for Global Severity Index (GSI) score. SPSS software, version 14 (SPSS Inc, Chicago, Il, USA) was used for analysis. Chi-square, Fisher’s exact test, t student, Mann–Whitney U, one-way ANOVA, and Kruskal-Wallis tests were used when appropriate. Logistic regression was used to estimate odds ratios for various determinants of students’ mental health.

**Results: **About half of the participants in the 2 groups were male (P = 0.63), and the mean age of the students in the reformed and non-reformed programs was 24.8 (1.97) and 24.7(1.80), respectively (P = 0.9). About 20% of participants in the non-reformed and less than 2% of those in the reformed program had GSI score of more than 0.7. Medical students in the reformed program had lower scores in total GSI and 9 its dimensions (P<0.001). The results obtained from the logistic regression analysis indicated that reformed curriculum and good economic status were significant independent variables contributing to decreased psychological distress (OR = 0.016 and 0.11, respectively).

**Conclusion: **The results revealed that curricular changes which were based on World Federation of Medical Education recommendation, could be associated with improvement in mental health status of medical students.

General medical degree (GMD) training usually causes significant psychological distress for physicians-in-training, especially in transition periods between preclinical, clerkship, and internship periods (1). However, there is little evidence on how GMD curricular changes leads to distress, what effects should be expected, and how the curriculum should be changed to alleviate distress (2). 

Dyrbye et al., in a systematic review, demonstrated higher prevalence of depression and anxiety in medical students, especially females, as compared to the general population and age-adjusted peers (2). Other studies on mental health of medical students revealed different levels of stress. The prevalence of probable mental disorders among medical students was reported to be 20% to 35% in developed countries (3-6) and 31% to 51.8% in Iran (7, 8). 

One major concern in the literature is that GMD curriculum should be changed periodically to meet the new health system needs (9). According to Edinburg Declaration in 1988, Iranian GMD curriculum has gone through extensive reform in Shahid Beheshti University of Medical Sciences in Tehran, the capital of Iran. The fundamental changes included enhancing students’ responsibility, critical appraisal in educational program, linkage between basic and clinical sciences, early clinical experience (horizontal integration), integration of physiopathology courses with clerkship (vertical integration), and more focus on community oriented medical education (10).

This study was conducted on 2 concurrent groups of medical students trained under either reformed or non-reformed GMD curriculum in the internship period in Shahid Beheshti University of Medical Sciences. This study was conducted to assess the effect of curricular changes in GMD program on mental health of medical students during internship.

## Materials and Methods

To examine the effect of new GMD educational curriculum on students’ mental health, all students in the reformed GMD program during internship were observed. Among 63 students, 60 accepted to participate in the study as the intervention group. Among 180 students in non-reformed GMD program, 120 were randomly selected as the control group. To avoid the effect of time on mental health, a unique opportunity was used: when the first group of students in the reformed GMD program started their internship, the students of previous non-reformed program were already in the internship stage, and thus the 2 groups were examined concurrently.

GMD curriculum of Shahid Beheshti University of Medical Sciences was completely revised in 2004. All the courses were made “organ based” and physiopathology courses were integrated in clerkship period. The contents were divided into core and non-core and courses to essentials and electives. In a student-centered approach, new educational technologies and methods, such as small group and project-based learning were applied to improve the quality of education. Community-based medical education was extended from the first to the last year of medical education period. Early patient contact, early community exposure, community-based risk assessment, and interventions and even office-based courses were developed. In Iran, GMD program takes 7 years and internship takes place in the last 18 months of the program; however, in the reformed GMD, internship period is extended to 2 years (10). 

Students were provided with a self-rated standard mental health questionnaire, Symptom Checklist-90-Revised (SCL-90-R), which is a 90-item self-report tool developed by Derogatis et al. in 1973 (11). It was developed to reflect the psychological symptom patterns of the community and respondents. The SCL-90-R was translated into the official language of Iran (Persian), which is understandable to almost every Iranian, and its validity and reliability were assessed in three independent studies (8, 12, 13). Correlation coefficient of the questionnaire based on pretest and posttest was 0.97 in the last study (13). Internal consistency for all dimensions of the questionnaire was more than 0.70. The sensitivity and specificity of the questionnaire according to psychiatric interview, compared to DSM III-R, was 0.94 and 0.98, respectively (12, 14). Each item in SCL-90-R is rated by the patient on a 5-point Likert scale. These points of stress are rated from 0 (none) to 4 (extreme) to measure the extent to which the respondent experienced the asked symptoms during the last 7 days. There are 7 items as additional questions in SCL-90-R, which are important for assessing clinical symptoms of the respondents. SCL-90-R consists of the following 9 primary symptom dimensions: somatization (SOM); obsessive-compulsive (O-C); interpersonal sensitivity (I-S); depression (DEP); anxiety (ANX); hostility (HOS); phobic anxiety (PHOB); paranoid ideation (PAR); and psychoticism (PSY). Raw scores were calculated by dividing the sum of the scores for a dimension by the number of items in that dimension. In this study, Global Severity Index (GSI) score and raw scores of SCL-90-R subscales were reported. Other questions on demographic factors (age, gender, marital status, and number of children) and socioeconomic status (partner’s degree and occupation, housing situation, owning a private car and cellphone) were included. There were 2 general questions on satisfaction of private life and study discipline: “Are you satisfied with your life overall?” and “Are you satisfied with your studying discipline?”

Similar to other studies, the GSI cut-off point for mental disorder was considered as 0.7, so those scoring 0.7 and above were designated as possible cases of mental disorder (8, 12, 15 and 16).

Students entered the study after providing informed consent. The questionnaires were anonymous and data were kept confidential. This study was performed in accordance with the declaration of Helsinki and was approved by the Ethical Committee of Shahid Beheshti University of Medical Sciences. 

All statistical analyses were performed using SPSS software, Version 14 (SPSS Inc, Chicago, Il, USA). Chi-square and Fisher’s exact test were used for categorical variables, and t student, Mann–Whitney U test, one-way ANOVA, and Kruskal-Wallis test were utilized for continuous variables. Multivariate analysis was conducted by logistic regression (Enter Method), and odds ratios were also calculated. P-value less than 0.05 was considered statistically significant.

## Results

Of the participants, 91 (50.8%) were male, 151 (83.9%) were single, and 169 (88.6%) had no children. There were statistically significant differences between reform and non-reform responders in marital status, number of children, type of residence, satisfaction of private life, and length of internship. Other sociodemographic characteristics are illustrated in Table 1.

Bivariate and multivariate analyses of the mean and standard deviation of the total GSI score and the 9 subscales of SCL-90-R questionnaire are demonstrated in Table 2. All the scores were significantly higher in the non-reform group. Analysis revealed that out of all students, 23 (12.8%) had GSI score of more than 0.7 (1.7% of the reformed and 19.2% of the non-reformed group).

Based on the logistic regression analysis, Nagelkerke R square was 39.7%. The results obtained from the logistic regression analysis indicated that type of GMD curriculum and economic status were significant independent variables in predicting probable mental disorder during internship. Medical students with good economic status who studied in the reform GMD program had lower GSI scores (Table 3).

**Table 1:** Demographic and Other Characteristics of the Medical Students Participant

	**Non-reformed N (%)**	**Reformed N (%)**	**Total N (%)**	**P- value**
Number	120 (66.7)	60 (33.3)	180 (100)	
Gender				0.63
Female	60 (50.4)	28 (46.7)	88 (49.2)	
Male	59 (49.6)	32 (53.3)	91 (50.8)	
Marital status				0.012
Single	97 (80.8)	54 (90)	151 (83.9)	
Married	22 (18.3)	6 (10)	28 (15.5)	
Divorced	1 (0.9)	0	1 (0.6)	
Number of children				<0.001
Zero	109 (92.4)	60 (100)	169 (88.6)	
One	7 (5.9)	0	7 (8.9)	
Two and more	2 (1.7)	0	2 (2.5)	
Literacy of spouse				0.06
Diploma	3 (8.8)	0	3 (7.5)	
BS	12 (35.3)	0	12 (30)	
MS	6 (17.6)	0	6 (15)	
Doctorate	13 (38.2)	6 (100)	19 (47.5)	
Job of the spouse				0.059
Governmental	9 (27.3)	0	9 (23.1)	
Non-governmental	8 (24.2)	0	8 (20.5)	
University student	11 (33.3)	6 (100)	17 (43.6)	
Housewife	5 (15.2)	0	5 (12.8)	
Type of residence				<0.001
Own personal	38 (34.5)	7 (11.7)	45 (26.5)	
Rented	13 (11.8)	1 (1.7)	14 (8.2)	
Dormitory	31 (28.2)	33 (55)	64 (37.6)	
With parents	28 (25.5)	19 (31.7)	47 (27.6)	
Owning private car				0.87
Yes	60 (51.3)	30 (50)	90 (50.8)	
No	57 (48.7)	30 (50)	87 (49.2)	
Owning cell phone				0.49
Yes	112 (95.7)	56 (93.3)	168 (94.9)	
No	5 (4.3)	4 (6.7)	9 (5.1)	
Economic status				0.06
Weak	16 (13.7)	16 (27.1)	32 (18.2)	
Fair	64 (54.7)	24 (40.7)	88 (50)	
Good	37 (31.6)	19 (32.2)	56 (31.8)	
Satisfaction with private life				0.03
Weak	18 (15)	19 (31.7)	37 (20.6)	
Fair	58 (48.3)	23 (38.3)	81 (45)	
Good	44 (36.7)	18 (30)	62 (34.4)	
Satisfaction with studying discipline				0.18
Weak	14 (11.7)	2 (3.3)	16 (8.9)	
Fair	46 (38.3)	25 (41.7)	71 (39.4)	
Good	60 (50)	33 (55)	93 (51.7)	
	**Mean (SD)**	**Mean (SD)**	**Mean (SD)**	**P value (CI)**
Age	24.8 (1.97)	24.7 (1.46)	24.8 (1.80)	0.97 (-0.51_0.53)
Length of internship (months)	10 (4.84)	4.2 (2.62)	7.6 (4.97)	<0.001 (4.46_7.18)
Minimum monthly shifts (days)	6.7 (2.16)	7.19 (0.79)	6.9 (1.62)	0.12 (-1.09_0.14)

**Table 2:** Scores of the Total GSI and 7 Scopes of SCL-90-R Questionnaire in the Reformed and Non-Reformed Groups in Medical Students

	**Non-reformed**	**Reformed**	**P-value**	**95% CI**
	**Mean (SD)**	**Mean (SD)**		
Total GSI	0.39 (0.27)	0.05 (0.04)	<0.001	0.28-0.38
Somatization	0.40 (0.30)	0.09 (0.23)	<0.001	0.22-0.38
Obsessive-compulsive	0.44 (0.30)	0.06 (0.17)	<0.001	0.31-0.45
Interpersonal sensitivity	0.39 (0.31)	0.07 (0.23)	<0.001	0.23-0.39
Depression	0.42 (0.33)	0.06 (0.15)	<0.001	0.28-0.42
Anxiety	0.37 (0.31)	0.07 (0.19)	<0.001	0.22-0.37
Hostility	0.40 (0.32)	0.03 (0.11)	<0.001	0.31-0.44
Phobic Anxiety	0.24 (0.30)	0.01 (0.06)	<0.001	0.17-0.28
Paranoid ideation	0.41 (0.30)	0.04 (0.09)	<0.001	0.31-0.43
Psychosis	0.32 (0.28)	0.02 (0.06)	<0.001	0.24-0.35

GSI: Global Severity Index

SCL-90-R: Symptom Checklist-90-Revised

**Table 3:** Outcome of the Logistic Regression Analysis of Related Variables and Recoded GSI Score in Medical Students

		**B**	**SE**	**OR (95% CI)**	**P- value**
Type of curriculum					
	Non-reform	4.156	1.399	63.82 (4.111-990.917)	0.003
	reform			1 (ref.)	
Economic status					
	Weak	2.169	1.082	8.75 (1.049-73.017)	0.045
	Fair	0.255	0.810	1.29 (0.264-6.316)	0.75
	Good			1 (ref.)	
Gender					
	Female	0.633	0.623	1.88 (0.555-6.390)	0.31
	Male			1 (ref.)	
Marital status					
	Married	1.097	0.805	1.46 (0.932-11.640)	0.23
	Single			1 (ref.)	
Type of residence					
	Rented	0.065	1.004	1.06 (0.149-7.644)	0.95
	Dormitory	-1.404	1.008	0.24 (0.034-1.772)	0.16
	With parents	-0.111	0.832	0.89 (0.175-4.567)	
	Owned			1 (ref.)	
Private car					
	No	-0.90	0.715	0.91 (0.225-3.712)	0.90
	Yes			1 (ref.)	
Mobile phone					
	No	1.193	1.244	3.29 (0.288-37.758)	0.33
	Yes			1 (ref.)	
Satisfaction with private life					
	Good	-1.24	1.291	0.29 (0.023-3.638)	0.33
	Fair	0.237	0.987	1.26(0.183-8.768)	0.81
	Weak			1 (ref.)	
Satisfaction with studying discipline					
	Good	-1.887	1.150	0.15 (0.016-1.443)	0.10
	Fair	-0.697	1.065	0.49 (0.062-4.015)	0.51
	Weak			1 (ref.)	

GSI: Global Severity Index

## Discussion

According to the results of the study, GSI scores of medical students in the reformed GMD program were lower than those in the non-reformed group. The possible explanation for this difference is lesser stress in students because of the structure and content of the reformed GMD program. Dividing courses to core and non-core and early exposure of students to the clinic and community through community-oriented courses in the reformed program might have been responsible for lowering the stress. Students in the reformed program were encouraged to concentrate on core courses, thus, the stress of non-core courses that need more effort may be decreased dramatically. Early clinical and community exposure could help the students to study more effectively and enter to clinical phase with lower stress. The results of this study agree with those of Kiessling et al, who showed that the reformed track provide more support, high quality of courses, and fulfillment of the students’ expectations (17). Godefrooij et al. also indicated that early patient contact seems to alleviate the shock of practice and prepare students for clinical courses (18).

Our results revealed that only 1 student (1.7%) had GSI score upper than the threshold; nearly 20% of the medical students in the non-reformed GMD program had GSI scores above the threshold. The results of non-reform program is in agreement with those of Nojomi et al. (12). However, our GSI score are lower than those reported in other studies in different countries (3-7, 19-23).This considerable difference, less than 2% in the reformed GMD and 20%- 40% in other studies, may be explained partly by curriculum changes in our study.

Compared to Iranian general population, the prevalence of probable mental disorder in the two groups in this study, especially the reformed group, was low (24). This difference was seen in other studies that compared Iranian students’ mental distress in various disciplines using SCL-90-R questionnaire. They reported GSI score in medical and non-medical students as 0.6 and 0.36, respectively (14, 25), The findings of Biro et al. study also confirmed that medical students had higher levels of stress and depression compared to non-medical undergraduates or peers (6).These findings raise a concern, as we need healthy students to promote mental health of the community(10). 

According to this study, there was no significant difference in GSI score between genders, this result is in line with Tabrizi-Zade et al. (7) and Sadeghian et al. studies in Iran (20), Omigbodun et al. (26) in Nigeria, and Sreeramareddy et al. in Nepal (4). However, our results disagree with some studies in Iran that showed female medical students had significantly higher scores (8, 12, 14, 19, 21 and 22). In the United States, in 2011, Childers et al., using SCL-90-R, showed that male students were significantly more stressed as compared to female students (3). 

Our results revealed no significant correlation between satisfaction with studying medicine and satisfaction with private life with GSI scores. In Nojomi et al. study, participants with good overall satisfaction, scored lower than other groups on all subscales of SCL-90-R, except for paranoia. There were significant associations between 6 subscales and satisfaction with medicine (12).

Logistic regression was used to control the differences between baseline characteristics of the study participants and possible confounders. Our finding showed an odds ratio of 63.82 (p = 0.003) for GSI score in the non-reformed group. However, other studies failed to find a significant relationship between curricular changes and psychological well-being of medical students (2).

The strength of our study was comparing the effect of two medical education programs on medical students’ mental health at the same time at the same university. This helped the researchers to avoid possible confounders of different educational environments at different times. 

## Limitation

On the other hand, a considerable limitation of major educational interventions, as in our study, is the impossibility of random allocation of students between two groups. One of the factors that could affect the findings of this study was baseline characteristics of the participants. Thus, the participants were compared regarding these characteristics. After adjustment, it was found that using logistic regression, type of curriculum, and economic status were independent predictors of GSI score. Another potential limitation was that the study was designed after the start of the non-reform internship period, so we did not have the opportunity to compare baseline mental status of the participants. As a result, we compared them once at the time of internship period. 

## Conclusion

The unique opportunity for comparing traditional and reformed GMD program in the same medical school showed that curricular changes could be associated with improvement in mental health status of medical students. This result recommends the same targeted curricular changes in medical schools to train healthier doctors for the community.
